# Annotations

**Published:** 1903-06-13

**Authors:** 


### ANNOTATIONS.

### Bad Business.

It has been the wise practice of the hospitals, and also of those responsible for the great central funds which raise money for hospitals, to avoid competition by arranging the dates of public events on behalf of hospitals each year so as to prevent any clashing. Of the 52 weeks in each year one in the middle of June has been assigned for years to the appeal on behalf of all the hospitals through the churches. By common consent this Hospital Sunday week has heretofore been left practically free from other appeals. This year, by coincidence or design, practically the whole of the week preceding Hospital Sunday is occupied with public efforts on behalf of individual hospitals. That much confusion and more annoyance should be caused to the public is only natural. How great the confusion is is shown by the fact that the Lord Mayor's letter on behalf of one hospital is headed, in the *Standard* of the 10th instant, "Hospital Sunday." This error arises no doubt from the fact that the Lord Mayor has usually written a letter for publication at this time on behalf of all the hospitals of London, and few could suppose that he would forestall his Hospital Sunday appeal by one on behalf of an individual hospital. The proceedings of the present week must bring matters to a crisis, and the Sunday Fund Council may be well advised to enlarge Law 9 of the constitution, so as to include cases where individual hospitals place their appeals in competition with that of the churches during the Hospital Sunday week. We are confident from the expressions of opinion which have reached us during the last few days that the hospitals immediately concerned are likely to be injured rather than helped by the proceedings in question, which from every point of view must prove to be bad business. The King made it a strong point in his letter to the people of London founding the King's Fund that all competition of the kind must be avoided. The wisdom, nay the necessity, of observing such a policy is obvious. The public nowadays is a well-educated public, and very properly resents the indiscretion which appears to thrust the claims of a particular institution continuously in front of every individual and collective effort in the same branch of charity.

### "Kissing the Book."

Is it not time that, with the advance of sanitary science, and the growing dread of infection, some more rational method of swearing to tell the truth in a court of law should be invented than the old one of kissing the Bible? When one reflects on the number of hands, more or less grimy, which have held, and the number of lips which have pressed the court copy of the New Testament, the idea of kissing these filthy boards becomes intolerable. In one place it is said that the Testament used in swearing witnesses was new in 1798, and has been kissed by 100,000 people! Yet persons who would object, and rightly, to using a serviette which had not passed through the laundress's hands since one person used it, are expected to press their lips to this book, and may be suspected of a disposition to commit perjury if they refuse. It is said that intending perjurers occasionally kiss the thumb which holds the book, instead of the book itself, and think that by this evasion they will escape the Divine punishment of their sin. There is something grotesque in this attempt to cheat the justice of Omniscience, but it is certain that these would-be perjurers have a better chance of escaping any infection that may lurk about the book than honester folks. But, for good or evil, superstition is now so dead that the man who means to lie in the witness-box will not be deterred by the dread of Divine punishment, whether he kiss the book or no, and therefore there is no reason why there should not be a change in administering the oath, which in the present form is much more likely to offend a truthful but fastidious witness, than to cause a liar to speak the truth. In Scotland, the witness, when taking the oath, holds up his right hand. This method is quite as impressive and quite as visible to the members of the court and such of the public as may be present, as bending the head to kiss a more or less dirty volume, and there is no such sanitary objection to it. Possibly English lawyers may be unwilling to borrow even so slight a detail of procedure from Scottish law, but if that be so, they should set to work to invent some method of making it a matter of conscience to a witness to speak the truth, without compelling him to resort to this antiquated and insanitary practice.

### The Common Cold.

There is no doubt that the ordinary nasal catarrh is a specific infectious disease. What we observe among domestic animals affords ample evidence of this. It is a familiar fact that a horse that has been wintered out, on being brought into a stable with others is most likely to develop a cold. The coachman will say it is because the unaccustomed warmth of the stable makes him "nesh." However, disinfection of the stable before bringing animals from grass is a true preventive of the symptoms of catarrh. What occurs among domestic animals we observe too among ourselves. Some source of infection must be present before it is possible to catch a cold. There are places where colds are unknown. The universal experience of Arctic and Antarctic explorers is that so long as the members of the expedition are in the polar regions they remain free from colds, but on return to the mainland or to settlements inhabited by those who are in frequent communication with the mainland, they nearly always at once suffer severe colds. The same is said to be true of the men in the observatory on the summit of Ben Nevis, though they live in clouds. Colds they never take, because there are no colds to catch, until the moment they descend to inhabited regions, then they catch severe ones directly. For over two centuries the classical St. Kilda cold has not ceased to interest learned men. On this remote and rocky island of the Western Hebrides, where some 100 inhabitants dwell, colds are unknown except after the arrival of a ship from the mainland, when all the inhabitants are seized with colds, even to the babe at the breast. Afterwards they seem to become to some extent immune, for many escape until the following year. The inhabitants affirm that those colds which are brought by boats from the large ports, Glasgow and Liverpool, are more severe than those brought from the Hebrides.

